# Multiomics Mapping Reveals Biological Insights Into the Muscle-to-Mental Axis During Aging

**DOI:** 10.1093/geroni/igaf122.4404

**Published:** 2025-12-31

**Authors:** Kaili Sun, Xucheng Wu, Hongwei Chen, Xueqing Jia, Weijing Gao, Xiaoyan Jiang, Xiaofeng Wang, Zuyun Liu

**Affiliations:** Zhejiang University, Hangzhou, Zhejiang, China (People’s Republic); Zhejiang University, Hangzhou, Zhejiang, China (People’s Republic); Zhejiang University, Hangzhou, Zhejiang, China (People’s Republic); Zhejiang university, Hangzhou, Zhejiang, China (People’s Republic); Zhejiang university, Hangzhou, Zhejiang, China (People’s Republic); Tongji University, Shanghai, Shanghai, China (People’s Republic); Fudan University, Shanghai, Shanghai, China (People’s Republic); Zhejiang University, Hangzhou, Zhejiang, China (People’s Republic)

## Abstract

Peripheral organs, including the skeletal muscle, play an important role in mental health. However, how skeletal muscle degeneration impacts mental disorders and the underlying mechanisms have not been fully understood. This study dissected the muscle-to-mental axis utilizing a multidimensional dataset of samples from the UK Biobank and the Rugao Ageing Study. Longitudinal analysis observed that skeletal muscle degeneration was associated with an increased risk of mental disorders, including depression and anxiety. This study mapped the multiomics signatures linked to muscle and mental disorders, pinpointing >2,000 statistically significant associations and 742 biomarkers, comprising 31 gut microbial species, 71 metabolites and 640 proteins. Notably, deleterious molecules (e.g., glycoprotein acetyls, growth differentiation factor 15, and urokinase plasminogen activator surface receptor) are known for their roles in inflammation, apoptosis and mitochondrial dysfunction. Conversely, protective molecules [e.g., valine (Val), leucine-rich repeat neuronal protein 1, and integrin alpha-11] are implicated in cellular homeostasis, neuroprotection and immune regulation. Further analysis revealed that the impact of skeletal muscle degeneration on mental disorders may occur through immunoinflammatory processes, and alterations in gut microbiome, metabolome and proteome. The multiomics integrative analysis identified an interaction network of key molecules (e.g., Val, T-cell surface glycoprotein CD4, and Paenibacillus thermotolerans) regulating muscle and mental disorders. Taken together, the multiomics mapping deepens our understanding of the muscle-to-mental axis during aging and reveals critical molecules as geroprotectors of multiple systems.

